# Emerging Use of Vaginal Laser to Treat Genitourinary Syndrome of Menopause for Breast Cancer Survivors: A Review

**DOI:** 10.3390/medicina59010132

**Published:** 2023-01-09

**Authors:** Nida Jugulytė, Guoda Žukienė, Daiva Bartkevičienė

**Affiliations:** 1 Faculty of Medicine, Vilnius University, LT-03101 Vilnius, Lithuania; 2 Clinic of Obstetrics and Gynaecology, Faculty of Medicine, Institute of Clinical Medicine, Vilnius University, LT-03101 Vilnius, Lithuania

**Keywords:** genitourinary syndrome of menopause, vulvovaginal atrophy, breast cancer survivor, vaginal laser

## Abstract

Breast cancer treatment, such as chemotherapy and endocrine therapy, can cause earlier and more sudden menopausal symptoms. Genitourinary syndrome of menopause (GSM) is one of the most bothersome side effects of breast cancer treatment, resulting in sexual dysfunction and impaired quality of life. GSM includes genital, urinary, and sexual symptoms. However, alleviating symptoms of GSM for breast cancer survivors may be challenging due to ineffectiveness, contraindications, and low adherence to treatment. The most recent data show the feasibility and safety of vaginal laser to treat GSM for breast cancer survivors. This narrative review provides the aspects of GSM in breast cancer patients, putting the focus on the efficacy and safety of vaginal laser therapy.

## 1. Introduction

Breast cancer is an important global health issue, as it is the most commonly diagnosed malignancy in the world [1]. Owing to advances in breast cancer screening and improved treatment, the current five-year survival rate is as high as 90% [2]. However, adjuvant therapy for breast cancer, including systemic chemotherapy and endocrine therapy, can cause undesirable symptoms that impair patients’ quality of life. One of the most bothersome side effects is genitourinary syndrome of menopause (GSM) [3], which is associated with impaired sexual function and lower quality of life [4,5].

According to the North American Menopause Society (NAMS), GSM, formerly known as vulvovaginal atrophy, is a syndrome characterized as a set of genital, urinary, and sexual symptoms caused by diminished estrogenic stimulation to the female genitourinary tract after the onset of menopause [6]. The main symptoms of GSM are vaginal dryness, irritation, burning, or itching; dyspareunia, decreased lubrication with sexual activity, or decreased arousal; and dysuria or increased urinary frequency [7]. The decline in estrogen level results in a thinner, drier, and less elastic vaginal epithelium. The changes due to estrogen withdrawal also include modified smooth muscle cells, expanded connective tissue, a decrease in collagen synthesis, and a reduction of blood vessels [8]. Epithelial thinning and lower glycogen impairs the homeostatic vaginal microenvironment leading to the decreased dominance of *Lactobacilli* and increased vaginal pH [9]. Since the female lower urinary tract also expresses estrogen receptors, a state of hypoestrogenism induces thinning of the urinary epithelium and reduces the strength of adjacent tissue [8]. It is to be noted that genitourinary symptoms can be chronic and progressive and usually do not improve over time [7].

Even though GSM usually develops in women after natural menopause, in reproductive age women, other reasons related to reduced estrogen levels may also provoke or worsen symptoms of GSM; these reasons include hyperprolactinemia during lactation, hypoestrogenism due to autoimmune disorders, as well as various pharmacological and iatrogenic causes, such as previously mentioned breast cancer treatment [10]. This means that younger women can also be at increased risk of developing GSM.

Alleviating the symptoms of GSM in women with a history of breast cancer is still an unmet need [3]. Nonhormonal vaginal gels may not provide the expected benefit; besides, women may find it difficult to apply vaginal gel two to three times per week [11]. Some patients are reluctant to use local estrogen as it may possibly be absorbed into the bloodstream and potentially stimulate neoplastic cells [12]. Moreover, hormone replacement therapy may be unsafe for breast cancer survivors. Fortunately, currently emerging vaginal laser therapy shows similar improvement in GSM symptoms compared to local estrogen treatment in healthy women after natural menopause [13]. In addition, there is an increasing number of studies demonstrating the feasibility of vaginal laser to treat GSM for breast cancer survivors [11,14,15,16,17,18,19,20,21,22,23,24,25]. Unfortunately, a survey of oncology health professionals reveals that only 3% of respondents recommend vaginal laser for treating GSM, while the main concerns of laser therapy are cost, efficacy, safety, availability, and a lack of knowledge [26].

The present narrative review aims to overview the peculiarities of GSM after breast cancer treatment, accentuating the importance of laser therapy.

## 2. Diagnosis and Assessment Tools of GSM

The evaluation of GSM is performed through a history and gynecological examination. Patients can report genital, urinary, and sexual symptoms. It is important to proactively inquire about symptoms suggestive of GSM because women are usually unwilling to discuss their urogenital or sexual complaints with their doctor [27]. A thorough anamnesis may reveal the presence and severity of urogenital symptoms and their influence on the patient’s sexual function and quality of life.

The physical examination can also be relevant in diagnosing GSM. An examination of the genitalia may reveal the loss of pubic hair, reduced mons pubis, fusion of the labia minora, narrow vaginal introitus, dry, thinned, pale and shiny vaginal mucosa, loss of elasticity of vaginal walls, shortened vagina or cervix, erythema, petechiae, and abnormal vaginal discharge. Several urinary signs, such as erythema and prominence of the urethral meatus, can be observed during the pelvic examination. In addition, vaginal pH can be measured, which is typically greater than 5.0 in GSM compared to about 3.8 to 4.5 in healthy vagina [6].

Several indexes are commonly used in research studies to evaluate the improvement of treatment for GSM; however, they are not required to make a diagnosis of GSM in clinical practice. These include the Visual Analogue Scale (VAS), the Vaginal Health Index (VHI), the Vaginal Maturation Index (VMI), and the Female Sexual Function Index (FSFI), the last of which has an adaptation for breast cancer patients (FSFI-BC) [28].

The severity and improvement of GSM symptoms in clinical trials are usually evaluated using the VAS. The VAS is a measurement tool for the self-report of intensity of symptoms.

Another subjective measuring instrument is the FSFI, which is used to evaluate female sexual function. It consists of six domains that include desire, subjective arousal, lubrication, orgasm, satisfaction, and pain. The total score ranges from 2 to 36, with higher scores implying better sexual functioning [29]. In 2015, a breast cancer specific adaptation of the FSFI was developed (FSFI-BC). The scale contains two additional subscales, which are changes after cancer and distress. According to the authors, the scale is suitable to routinely screen breast cancer patients for sexual dysfunction in clinical and research settings [30].

The VHI evaluates five parameters, which are vaginal elasticity, vaginal secretions, pH, epithelial mucous membrane, and tissue hydration. By assigning a single score to each element, it is possible to determine the degree of vaginal atrophy. The total score varies from 5 to 25, with lower scores indicating more severe atrophy [31]. It is noteworthy to mention that only one variable of the scale, which is vaginal pH, can be considered objective, so some authors express doubts whether or not the VHI is an objective measuring tool [28].

The VMI is an objective assessment instrument which categorizes the ratio of three vaginal epithelial cell types (parabasal, intermediate, and superficial) present on specimens obtained from the lateral upper vaginal walls. The higher proportion of parabasal cells is associated with hypoestrogenism and atrophy [31]. However, the VMI is not commonly used in most clinical trials studying GSM [28].

Overall, as stated in a review by Weber et al., subjective and objective assessment tools for vulvovaginal atrophy should be combined in both clinical and research settings. It is also noted that subjective assessment is the first priority in clinical practice, whereas an objective assessment is recommended in the research setting [32].

## 3. The Impact of Breast Cancer Treatment

### 3.1. Chemotherapy

One of the breast cancer treatment options is systemic chemotherapy. Systemic neoadjuvant chemotherapy provides greater surgical options, as it may make inoperable tumors operable by reducing tumor size as well as downstaging the disease. Adjuvant systemic therapy with cytotoxic agents may improve overall survival by eradicating the remaining deposits of disease along with reducing the rates of recurrence [33]. However, chemotherapy can have a significant negative effect on ovarian function and may lead to treatment-induced ovarian function suppression (OFS) [34]. The reason for this is the destruction of ovarian follicles due to the toxic chemotherapy effect to granulosa cells that surround an immature oocyte as well as oocytes themselves; however, the specific molecular mechanism related to the damage to ovaries during chemotherapy is still not fully explored [35]. Since reproductive steroid hormones are produced by follicles, the loss of the follicular reserve leads to decreased levels of estrogen. OFS occurs as transient or permanent amenorrhea along with menopausal symptoms, one of which is the GSM [36].

### 3.2. Endocrine Therapy

The vast majority of breast cancers are hormone-positive, as up to 77% of tumors show the overexpression of estrogen receptors (ERs) and/or progesterone receptors (PRs) [37]. The standard of care for women with ER-positive breast cancer is hormone therapy for 5–10 years [38]. The purpose of this therapy is to decrease the estrogen induced stimulation of neoplastic cells [39] either by reducing the effect of endogenous estrogen at the receptor level or by suppressing estrogen synthesis. Premenopausal patients with hormone-positive breast cancer are given tamoxifen or gonadotropin-releasing hormone (GnRH) analogs, while tamoxifen and aromatase inhibitors (AIs) are both indicated in postmenopausal women [40]. Since the female genitourinary tract is rich with estrogen receptors, estrogen deprivation associated with endocrine therapy may result in genitourinary atrophy [41]. However, various classes of endocrine therapy have different mechanisms of action in the vaginal tract; consequently, the frequency and severity of side effects varies significantly among breast cancer patients depending on the class of drug used.

#### 3.2.1. Tamoxifen

Tamoxifen is a selective estrogen receptor modulator (SERM) and it competes with estrogen to bind to the estrogen receptors in normal breast and breast cancer cells; hence, this antiestrogenic activity has an antitumor effect [42]. The reason for the increased incidence of GSM symptoms in tamoxifen-treated patients is mainly the blockage of circulating estrogens [43]. On the other hand, tamoxifen may contrarily improve existing vaginal dryness, as it enhances vaginal secretions due to the estrogenic effect on tissues other than breast [44].

#### 3.2.2. Aromatase Inhibitors

The mechanism of action of AIs is to inhibit an enzyme involved in the biosynthesis of estrogen from androgens [45]. Therefore, the inhibition of aromatase leads to decreased physiological concentrations of estrogen, and makes AIs an essential therapeutic strategy against endocrine-responsive breast cancer [46]. AIs influence on severe estrogen insufficiency as well as potential local inhibition of aromatase in vaginal tissue increases the severity of GSM symptoms. It is suggested that AIs result in a decrease of proliferation in the vaginal epithelium, which correlates with vaginal atrophy severity scores and vaginal pH [47]. It has been noted that AIs cause vaginal dryness and sexual dysfunction more often than tamoxifen [43,48,49,50].

## 4. Prevalence of GSM among Breast Cancer Survivors

Genitourinary symptoms are highly prevalent, affecting up to 84% of postmenopausal women [6]. The GSM symptom burden is even higher in patients following breast cancer treatment largely due to iatrogenic reasons as well as hesitation to use conventional remedies [51]. In surveys and cross-sectional studies, 35% to 91% of breast cancer survivors reported at least one GSM symptom [48,52,53,54,55,56,57]. Vaginal dryness and dyspareunia are the most frequently reported symptoms of GSM among breast cancer survivors, although severity and frequency of symptoms varies [52,53,54,55]. The prevalence of GSM symptoms depends on several factors, such as menopausal status and breast cancer treatment, including current adjuvant hormone therapy. With regard to menopause status, a cohort study of breast cancer survivors showed that vaginal dryness and dyspareunia were more prevalent in postmenopausal compared to premenopausal women (61.5% vs. 23.4%, 38.7% vs. 15.9%, respectively), and the severity of these symptoms was significantly higher among postmenopausal women [58]. Opposing results were found in a survey on breast cancer patients after chemotherapy and hormonotherapy, as 42% of premenopausal and 19% of postmenopausal women reported vaginal dryness [59]. With regard to endocrine therapy, several studies have demonstrated that AIs-treated patients report GSM symptoms more often than tamoxifen-treated women [48,49,50,60], but vaginal discharge is mostly attributed to tamoxifen [49,60].

## 5. Therapeutic Options for GSM in Breast Cancer Survivors

NAMS recommends nonhormonal vaginal lubricants and moisturizers, low-dose vaginal estrogens, vaginal dehydroepiandrosterone (DHEA) inserts, oral ospemifene, and oral hormone therapy in order to alleviate GSM symptoms for postmenopausal women. However, the management of GSM in women with a history of breast cancer can be challenging because estrogen-based products may increase the risk of breast cancer recurrence [6]. According to the American College of Obstetricians and Gynecologists (ACOG), low-dose vaginal estrogen, DHEA or testosterone may be used in this specific population if urogenital symptoms persist after a trial of nonhormonal treatments [61]. Nevertheless, both nonhormonal and local hormonal treatments have some disadvantages. Nonhormonal vaginal gels or moisturizers have to be applicated multiple times per week, which can compromise the long-term compliance with treatment [11], whereas the probability of increased cancer recurrence is still considered a barrier to the prescribing of vaginal hormonal therapy among a lot of oncologists [26,62]. Innovative laser therapy has emerged recently, and is a promising therapeutic approach for GSM.

### 5.1. Laser Types and Mechanism of Action

The two types of lasers that have been most investigated for alleviating the symptoms of GSM are the fractional microablative CO_2_ laser and the non-ablative erbium:YAG (Er:YAG) laser. These two lasers differ in characteristics, such as active medium, wavelength, and absorption by water. A fractional microablative CO_2_ laser uses a gas medium and delivers pulses at a wavelength of 10,600 nm. It is highly absorbed by water, so the depth of penetration is determined by the water content of the tissue. Due to the small diameter beam and pulsed instead of continuous energy, the superficial action of the laser and a less deep thermal damage are provided. The Er:YAG laser uses a solid medium and creates heat pulses at a wavelength of 2940 nm. Seeing that this wavelength is close to the peak absorption of water, the Er:YAG laser achieves more focused and deeper heating without ablation or overheating of superficial layers of the mucosa. The ability to coagulate is lower in the Er:YAG laser compared to the CO_2_ laser, which means that there is a higher probability of bleeding during treatment [63,64,65].

### 5.2. Morphological Changes of the Vaginal Tissue after Laser Therapy

In spite of the dissimilarity of the two laser types, the fundamental effect is assumed to be neocollagenesis, elastogenesis and neoangiogenesis, stimulating tissue restructuring and rejuvenation [65]. A sudden elevation in temperature in the vaginal mucosa provokes changes in cell metabolism by inducing the production of the heat shock proteins. Specific subtypes of the heat shock proteins promote the action of Transforming growth factor β (TGF-β) in the fibrogenic process. Fibroblasts are crucial for the production of a new extracellular matrix, collagen, and elastic fibers. This cascade, which lasts for thirty days, results in a regenerative and remodeling effect of vaginal tissue [64]. According to an ex vivo histological study, a microablative CO_2_ laser can contribute to a restructuring of vaginal connective tissue without damaging adjacent tissue, thereby ascertaining the restoration of atrophic vaginal tissue to a premenopausal state. Under an electron microscope, a highly represented rough endoplasmic reticulum, a well-developed Golgi complex, and compact bundles of renewing collagen fibers are observable. These features are linked to a fibroblast stimulation with the production of collagen and other components of the extracellular matrix. In other words, the use of an intravaginal laser leads to neocollagenesis and reconstruction of the trabecular architecture of the collagen itself, contributing to the amelioration of strength and elasticity of vaginal tissue [66]. This perception is supported by Zerbinati et al.’s study which determined the CO_2_ laser induced microscopic and ultrastructural modifications of vaginal mucosa. This study reports a metabolic reactivation of the components within the connective tissue as well as a new production of glycogen and acidic mucins within the epithelium of the vaginal mucosa following microablative CO_2_ laser treatment. In addition, a thicker squamous stratified epithelium, formed by 20–40 cell layers, could be seen under a light microscope at 1-month and also at 2-months follow-up. A thick epithelium ensures the differentiation of cells and superficial shedding [67]. Furthermore, a morphometric analysis of atrophic vaginal mucosa specimens demonstrated an increase in the number of blood capillaries and their volume density, along with an expansion of the epithelial layer thickness under the Er:YAG laser exposure. In addition, an analysis of vaginal biopsy specimens showed no neutrophilic and eosinophilic infiltration, and no signs of inflammatory reaction were indicated [68]. All of these morphological changes suggest a therapeutic vector in managing GSM symptoms.

### 5.3. Efficacy of Laser Therapy

Table 1 shows the results of the thirteen studies that investigated the efficacy and safety of laser therapy for GSM symptoms among patients after breast cancer treatment. Almost all included studies were single-arm with no comparison group, except for one study [23] in which women with GSM symptoms but no history of breast cancer comprised a comparison group. Sample sizes were rather small in all included studies, ranging from 16 to 135. In total, 502 breast cancer survivors participated in the studies. The majority of studies [14,15,16,19,20,21,24,25] administered three cycles of vaginal laser treatment every 30 days; however, other approaches of laser administration were also used as the optimal number of cycles has yet to be defined.

Considering short-term results, there was a significant improvement of VAS [14,15,16,17,19,21,22,23], VHI [11,15,16,17,18,20,22,23], and FSFI [17,21,22,24] scores before and after laser therapy. Unfortunately, none of these studies used the VMI, which is an objective assessment tool.

Four studies [16,23,24,25] evaluated the long-term sustainability of the improvement of GSM symptoms in breast cancer survivors, and the results are promising. Gambacciani et al. [16] reported a maintained effect of laser therapy for at least 12 to 18 months. Siliquini et al. [23] noted a progressive and long-lasting improvement of up to 12 months after the end of treatment, showing the vaginal laser to be an effective option in BCS. Veron et al. [24] conducted a study with 18 months follow-up. It showed maintained improvement in sexual and urinary function, although the observed effect decreased after 6 months. Quick et al. [25] suggested the potential long-term benefit of laser therapy as sexual function remained improved two years after treatment completion. There was a slight increase in the mean VAS score from the 4 week follow-up to the 2 year follow-up, but it was not statistically significant.

Siliquini et al. [23] included the comparison group of women with GSM symptoms and without a history of breast cancer. This study revealed that improvement was obtained more slowly among breast cancer survivors than among women with no history of breast cancer receiving the same laser treatment for GSM symptoms. Significantly higher levels of dyspareunia and vaginal dryness were observed from the third laser session until the six months follow-up visit in breast cancer survivors. In addition, the VHI was within normal limits after only one laser session in the comparison group, whereas among breast cancer survivors the VHI value was normal after two laser sessions.

In regard to the comparison of the two types of intravaginal laser, there are currently no studies published comparing the effectiveness of the microablative CO_2_ and Er:YAG lasers for the management of symptoms of GSM in women either with breast cancer or without cancer. However, as both types of laser show the alleviation of GSM symptoms, it seems that there is an equivalence of these two laser technologies.

It is worthy of note that even though numerous before and after studies show promising results, there is still a need for randomized, sham-controlled studies to draw confident conclusions about the efficacy of laser therapy in managing GSM in breast cancer survivors.

### 5.4. Safety of Laser Therapy

Concerning the safety of laser therapy, all papers were quite consistent, as no severe adverse events were observed during treatment. Several patients reported discomfort or pain related to probe insertion [15,19,24] and mild to moderate bleeding within 24 h of receiving treatment [11,24]. There was one case of vaginal candidiasis and one case of acute cystitis reported [20]. It is worthy of mention that three women had abnormal Pap smears (two had low-grade squamous intraepithelial lesions (LSIL) and one had a high-grade squamous intraepithelial lesion (HSIL)) during follow-up. Even though there are currently no data on a possible relationship between vaginal laser application and HPV infection, the contribution of laser therapy cannot be ruled out in the emergence of HPV-linked lesions [24].

## 6. Conclusions

GSM in breast cancer survivors is a serious and prevalent issue, as breast cancer is the most commonly diagnosed female cancer worldwide. GSM negatively affects women’s sexuality and general quality of life; therefore, the treatment of GSM is of great importance. However, the appropriate treatment for patients with a history of breast cancer is still an inadequately addressed problem. A treatment with a vaginal laser can lead to rejuvenation and restructuring of the vaginal tissue. Clinical studies showed a statistically significant and sustained alleviation of the GSM symptoms along with improvement in sexual function. In addition, no severe adverse events were recorded, making laser therapy a feasible and safe method to treat GSM for breast cancer survivors.

## Figures and Tables

**Table 1:** Results of the studies of laser therapy for GSM in BC survivors.

Authors, Year	Design	Type of Laser	Participants	Mean Age, Years	No of Patients	Sessions	Duration of Study	Measured Outcome	Outcome	Conclusion
Pagano et al., 2016 [14]	Retrospective study	Microablative CO_2_	Women with hormone-positive BC with VVA	42	26	3 cycles every 30 to 40 days	3 months	VAS	Reduced vaginal dryness, itching and dyspareunia (*p* < 0.0001)	Significant improvement of VVA symptoms in women affected by hormone-driven BC
Pieralli et al., 2016 [15]	Single-arm, prospective descriptive study	Microablative CO_2_	BC survivors with VVA	53.3	50	3 cycles every 30 days	11 months	VAS, VHI	Reduced dyspareunia (*p* < 0.0001). Higher VHI (*p* < 0.0001)	Feasible and effective treatment for VVA dyspareunia in BC survivors
Gambacciani et al., 2017 [16]	Single-arm, prospective study	Erbium YAG	Postmenopausal BC survivors with GSM	50.8	43	3 cycles every 30 days	18 months	VAS, VHI	Reduced vaginal dryness (*p* < 0.01), dyspareunia (*p* < 0.01). Higher VHI (*p* < 0.01)	Effective and safe treatment of GSM in BC survivors. Effects sustained for at least 12 to 18 months
Becorpi et al., 2018 [17]	Single-arm, prospective study	Microablative CO_2_	Postmenopausal BC survivors with VVA	58.2	20	2 cycles every 30 days	2 months	VAS, VHI, FSFI	Reduced vaginal dryness (*p* = 0.002), itching (*p* = 0.012), dyspareunia (*p* = 0.006). Higher VHI (*p* = 0.000). Higher FSFI (*p* = 0.003)	Effective treatment in postmenopausal BC survivors
Mothes et al., 2018 [18]	Retrospective study	Erbium YAG	BC survivors with GSM after pelvic organ prolapse surgery	71	16	1 cycle	6 weeks	VHI	Higher VHI (*p* = 0.01)	Simple, effective and safe treatment in postmenopausal BC survivors with atrophy-related complaints
Pagano et al., 2018 [19]	Retrospective study	Erbium YAG	BC survivors with VVA	44	82	3 cycles every 30 days	3 months	VAS	Reduced vaginal dryness, itching, dyspareunia and dysuria (*p* < 0.001)	Effective and safe treatment in BC patients with iatrogenic menopause
Areas et al., 2019 [20]	Open, prospective, therapeutic intervention study	Erbium YAG	Postmenopausal BC survivors with GSM symptoms	53.7	24	3 cycles every 30 days	3 months	VHI	Higher VHI (*p* < 0.001)	Improvement in sexual function and vaginal atrophy in postmenopausal BC survivors
Pearson et al., 2019 [21]	Single-arm, prospective study	Microablative CO_2_	BC survivors with VVA and with spontaneous or induced menopause	55	26	3 cycles every 30 days	12 weeks	VAS, FSFI	Reduced vaginal dryness, dysuria, dyspareunia (*p* < 0.001), itching (*p* < 0.01). Higher FSFI (*p* < 0.001)	Improvement in VVA symptoms and sexual function
Hersant et al., 2020 [11]	Single-arm, prospective study	Microablative CO_2_	BC survivors with VVA	56.1	20	2 cycles	6 months	VHI	Higher VHI (*p* < 0.0001)	Effective and safe method to improve the trophicity and decrease vaginal dryness in women with VVA after BC therapy
Salvatore et al., 2021 [22]	Single-arm, prospective study	Microablative CO_2_	BC survivors with VVA and who currently were or had been on endocrine therapy	57.9	40	5 cycles every 4 weeks	20 weeks	VAS, VHI, FSFI	Reduced vaginal dryness, itching, dyspareunia, dysuria (*p* < 0.001). Higher VHI (*p* < 0.05). Higher FSFI (*p* < 0.05)	Safe and effective in treating VVA symptoms in women with a history of BC
Siliquini et al., 2021 [23]	Retrospective study	Microablative CO_2_	Postmenopausal BC survivors with GSM; postmenopausal healthy women with GSM	60.6 (BC survivors); 58.4 (healthy women)	45 (BC survivors); 90 (healthy women)	3 cycles	12 months	VAS, VHI	Reduced vaginal dryness and dyspareunia (*p* < 0.05). Higher VHI (*p* < 0.001)	Long-term improvement in GSM symptoms in BC survivors. Improvement is slower among BC survivors than in healthy women undergoing the same treatment
Veron et al., 2021 [24]	Single-arm, prospective study	Microablative CO_2_	Postmenopausal BC survivors with GSM	56.5	46	3 cycles every 30 days	18 months	FSFI	Higher FSFI (*p* = 0.01)	Effective treatment on the long-term on VVA symptoms among BC survivors
Quick et al., 2022 [25]	Single-arm study	Microablative CO_2_	BC survivors with GSM	57.4	64	3 cycles every 30 days	2 years	VAS, FSFI	No difference in VAS score between the 4-week follow-up and 2-year follow-up (*p* = 0.15). FSFI remained improved at the 2-year follow-up	Sustained improvement in sexual function two years after treatment completion

BC, breast cancer; VAS, visual analogue scale; VHI, vaginal health index; FSFI, female sexual function index; GSM, genitourinary syndrome of menopause; VVA, vulvovaginal atrophy.

## Data Availability

Data sharing is not applicable to this article.

## References

[1] Wilkinson L., Gathani T. (2022). Understanding breast cancer as a global health concern. Br. J. Radiol..

[2] Siegel R.L., Miller K.D., Jemal A. (2020). Cancer statistics, 2020. CA Cancer J. Clin..

[3] Lubián López D.M. (2022). Management of genitourinary syndrome of menopause in breast cancer survivors: An update. World J. Clin. Oncol..

[4] Nappi R.E., Palacios S., Particco M., Panay N. (2016). The REVIVE (REal Women’s VIews of Treatment Options for Menopausal Vaginal ChangEs) survey in Europe: Country-specific comparisons of postmenopausal women’s perceptions, experiences and needs. Maturitas.

[5] Nappi R.E., Palacios S., Bruyniks N., Particco M., Panay N. (2019). Investigators on behalf of the ES. The burden of vulvovaginal atrophy on women’s daily living: Implications on quality of life from a face-to-face real-life survey. Menopause.

[6] North American Menopause Society (2020). The 2020 genitourinary syndrome of menopause position statement of The North American Menopause Society. Menopause.

[7] Portman D.J., Gass M.L.S., Panel on behalf of the VATCC (2014). Genitourinary syndrome of menopause: New terminology for vulvovaginal atrophy from the International Society for the Study of Women’s Sexual Health and The North American Menopause Society. Menopause.

[8] Lev-Sagie A. (2015). Vulvar and Vaginal Atrophy: Physiology, Clinical Presentation, and Treatment Considerations. Clin. Obstet. Gynecol..

[9] Amabebe E., Anumba D.O.C. (2018). The Vaginal Microenvironment: The Physiologic Role of Lactobacilli. Front. Med..

[10] Gandhi J., Chen A., Dagur G., Suh Y., Smith N., Cali B., Khan S.A. (2016). Genitourinary syndrome of menopause: An overview of clinical manifestations, pathophysiology, etiology, evaluation, and management. Am. J. Obstet. Gynecol..

[11] Hersant B., Werkoff G., Sawan D., Sidahmed-Mezi M., Bosc R., La Padula S., Kalsoum S., Ouidir N., Meningaud J.-P., Belkacemi Y. (2020). Carbon dioxide laser treatment for vulvovaginal atrophy in women treated for breast cancer: Preliminary results of the feasibility EPIONE trial. Ann. Chir. Plast. Esthétique.

[12] Santen R.J., Mirkin S., Bernick B., Constantine G.D. (2019). Systemic estradiol levels with low-dose vaginal estrogens. Menopause.

[13] Jang Y.C., Leung C.Y., Huang H.L. (2022). Comparison of Severity of Genitourinary Syndrome of Menopause Symptoms After Carbon Dioxide Laser vs Vaginal Estrogen Therapy: A Systematic Review and Meta-analysis. JAMA Netw. Open.

[14] Pagano T., De Rosa P., Vallone R., Schettini F., Arpino G., De Placido S., Nazzaro G., Locci M., De Placido G. (2016). Fractional microablative CO_2_ laser for vulvovaginal atrophy in women treated with chemotherapy and/or hormonal therapy for breast cancer: A retrospective study. Menopause.

[15] Pieralli A., Fallani M.G., Becorpi A., Bianchi C., Corioni S., Longinotti M., Tredici Z., Guaschino S. (2016). Fractional CO_2_ laser for vulvovaginal atrophy (VVA) dyspareunia relief in breast cancer survivors. Arch. Gynecol. Obstet..

[16] Gambacciani M., Levancini M. (2017). Vaginal erbium laser as second-generation thermotherapy for the genitourinary syndrome of menopause: A pilot study in breast cancer survivors. Menopause.

[17] Becorpi A., Campisciano G., Zanotta N., Tredici Z., Guaschino S., Petraglia F., Pieralli A., Sisti G., De Seta F., Comar M. (2018). Fractional CO_2_ laser for genitourinary syndrome of menopause in breast cancer survivors: Clinical, immunological, and microbiological aspects. Lasers Med. Sci..

[18] Mothes A.R., Runnebaum M., Runnebaum I.B. (2018). Ablative dual-phase Erbium:YAG laser treatment of atrophy-related vaginal symptoms in post-menopausal breast cancer survivors omitting hormonal treatment. J. Cancer Res. Clin. Oncol..

[19] Pagano T., De Rosa P., Vallone R., Schettini F., Arpino G., Giuliano M., Lauria R., De Santo I., Conforti A., Gallo A. (2018). Fractional microablative CO_2_ laser in breast cancer survivors affected by iatrogenic vulvovaginal atrophy after failure of nonestrogenic local treatments: A retrospective study. Menopause.

[20] Arêas F., Valadares A.L.R., Conde D.M., Costa-Paiva L. (2019). The effect of vaginal erbium laser treatment on sexual function and vaginal health in women with a history of breast cancer and symptoms of the genitourinary syndrome of menopause: A prospective study. Menopause.

[21] Pearson A., Booker A., Tio M., Marx G. (2019). Vaginal CO_2_ laser for the treatment of vulvovaginal atrophy in women with breast cancer: LAAVA pilot study. Breast Cancer Res. Treat..

[22] Salvatore S., Nappi R.E., Casiraghi A., Ruffolo A.F., Degliuomini R., Parma M., Maggiore U.L.R., Athanasiou S., Candiani M. (2021). Microablative Fractional CO_2_ Laser for Vulvovaginal Atrophy in Women with a History of Breast Cancer: A Pilot Study at 4-week Follow-up. Clin. Breast Cancer.

[23] Siliquini G.P., Bounous V.E., Novara L., Giorgi M., Bert F., Biglia N. (2021). Fractional CO₂ vaginal laser for the genitourinary syndrome of menopause in breast cancer survivors. Breast J..

[24] Veron L., Wehrer D., Annerose-Zéphir G., Suciu V., Delaloge S., Pistilli B., Chaltiel D., Pautier P. (2021). Effects of local laser treatment on vulvovaginal atrophy among women with breast cancer: A prospective study with long-term follow-up. Breast Cancer Res. Treat..

[25] Quick A.M., Hundley A., Evans C., Stephens J.A., Ramaswamy B., Reinbolt R.E., Noonan A.M., Van Deusen J.B., Wesolowski R., Stover D.G. (2022). Long-Term Follow-Up of Fractional CO_2_ Laser Therapy for Genitourinary Syndrome of Menopause in Breast Cancer Survivors. J. Clin. Med..

[26] Pearson A., Dhillon H.M., Kiely B.E. (2021). Genitourinary symptoms in women with breast cancer: What do oncology health professionals think and do about them?. Breast Cancer.

[27] Kingsberg S.A., Krychman M., Graham S., Bernick B., Mirkin S. (2017). The Women’s EMPOWER Survey: Identifying Women’s Perceptions on Vulvar and Vaginal Atrophy and Its Treatment. J. Sex. Med..

[28] Mension E., Alonso I., Tortajada M., Matas I., Gómez S., Ribera L., Ros C., Anglès-Acedo S. (2021). Genitourinary Syndrome of Menopause Assessment Tools. J.-Life Health.

[29] Rosen R., Brown C., Heiman J., Leiblum S., Meston C., Shabsigh R., Ferguson D., D’Agostino R. (2000). The Female Sexual Function Index (FSFI): A multidimensional self-report instrument for the assessment of female sexual function. J. Sex. Marital Ther..

[30] Bartula I., Sherman K.A. (2015). Development and validation of the Female Sexual Function Index adaptation for breast cancer patients (FSFI-BC). Breast Cancer Res. Treat..

[31] Nappi R.E., Martini E., Cucinella L., Martella S., Tiranini L., Inzoli A., Brambilla E., Bosoni D., Cassani C., Gardella B. (2019). Addressing Vulvovaginal Atrophy (VVA)/Genitourinary Syndrome of Menopause (GSM) for Healthy Aging in Women. Front. Endocrinol..

[32] Weber M.A., Limpens J., Roovers J.P.W.R. (2015). Assessment of vaginal atrophy: A review. Int. Urogynecology J..

[33] Fisusi F.A., Akala E.O. (2019). Drug Combinations in Breast Cancer Therapy. Pharm. Nanotechnol..

[34] Lambertini M., Del Mastro L., Viglietti G., Pondé N.F., Solinas C., de Azambuja E. (2017). Ovarian Function Suppression in Premenopausal Women with Early-Stage Breast Cancer. Curr. Treat Options Oncol..

[35] Kim S., Kim S.W., Han S.J., Lee S., Park H.T., Song J.Y., Kim T. (2021). Molecular Mechanism and Prevention Strategy of Chemotherapy- and Radiotherapy-Induced Ovarian Damage. Int. J. Mol. Sci..

[36] Cosgrove C.M., Salani R. (2019). Ovarian effects of radiation and cytotoxic chemotherapy damage. Best Pract. Res. Clin. Obstet. Gynaecol..

[37] Yip C.H., Rhodes A. (2014). Estrogen and progesterone receptors in breast cancer. Future Oncol..

[38] Loibl S., Poortmans P., Morrow M., Denkert C., Curigliano G. (2021). Breast cancer. Lancet.

[39] Drăgănescu M., Carmocan C. (2017). Hormone Therapy in Breast Cancer. Chirurgia.

[40] Reinbolt R.E., Mangini N., Hill J.L., Levine L.B., Dempsey J.L., Singaravelu J., Koehler K.A., Talley A., Lustberg M.B. (2015). Endocrine Therapy in Breast Cancer: The Neoadjuvant, Adjuvant, and Metastatic Approach. Semin. Oncol. Nurs..

[41] Sousa M.S., Peate M., Jarvis S., Hickey M., Friedlander M. (2017). A clinical guide to the management of genitourinary symptoms in breast cancer survivors on endocrine therapy. Ther. Adv. Med. Oncol..

[42] Andrahennadi S., Sami A., Manna M., Pauls M., Ahmed S. (2021). Current Landscape of Targeted Therapy in Hormone Receptor-Positive and HER2-Negative Breast Cancer. Curr. Oncol..

[43] Hale M.J., Howell A., Dowsett M., Cuzick J., Sestak I. (2020). Tamoxifen related side effects and their impact on breast cancer incidence: A retrospective analysis of the randomised IBIS-I trial. Breast.

[44] Lester J., Pahouja G., Andersen B., Lustberg M. (2015). Atrophic Vaginitis in Breast Cancer Survivors: A Difficult Survivorship Issue. J. Pers. Med..

[45] Kang H., Xiao X., Huang C., Yuan Y., Tang D., Dai X., Zeng X. (2018). Potent aromatase inhibitors and molecular mechanism of inhibitory action. Eur. J. Med. Chem..

[46] Avvaru S.P., Noolvi M.N., Aminbhavi T.M., Chkraborty S., Dash A., Shukla S.S. (2018). Aromatase Inhibitors Evolution as Potential Class of Drugs in the Treatment of Postmenopausal Breast Cancer Women. Mini-Rev. Med. Chem..

[47] Kallak T.K., Baumgart J., Göransson E., Nilsson K., Poromaa I.S., Stavreus-Evers A. (2014). Aromatase inhibitors affect vaginal proliferation and steroid hormone receptors. Menopause.

[48] Panjari M., Bell R.J., Davis S.R. (2011). Sexual Function after Breast Cancer. J. Sex. Med..

[49] Cella D., Fallowfield L.J. (2008). Recognition and management of treatment-related side effects for breast cancer patients receiving adjuvant endocrine therapy. Breast Cancer Res. Treat..

[50] Baumgart J., Nilsson K., Evers A.S., Kallak T.K., Poromaa I.S. (2013). Sexual dysfunction in women on adjuvant endocrine therapy after breast cancer. Menopause.

[51] Jasra B., Samiian L., Kaunitz A.M. (2019). Breast Cancer and the Obstetrician-Gynecologist: A Focus on Screening, Risk Assessment and Treatment of Survivors With Genitourinary Syndrome of Menopause. Clin. Obstet. Gynecol..

[52] Nappi R.E., Palacios S., Bruyniks N., Particco M., Panay N. (2021). The European Vulvovaginal Epidemiological Survey (EVES). Impact of history of breast cancer on prevalence, symptoms, sexual function and quality of life related to vulvovaginal atrophy. Gynecol. Endocrinol..

[53] Peate M., Saunders C., Cohen P., Hickey M. (2021). Who is managing menopausal symptoms, sexual problems, mood and sleep disturbance after breast cancer and is it working? Findings from a large community-based survey of breast cancer survivors. Breast Cancer Res. Treat..

[54] Sbitti Y., Kadiri H., Essaidi I., Fadoukhair Z., Kharmoun S., Slimani K., Ismaili N., Ichou M., Errihani H. (2011). Breast cancer treatment and sexual dysfunction: Moroccan women’s perception. BMC Womens Health.

[55] Stabile C., Goldfarb S., Baser R.E., Goldfrank D.J., Abu-Rustum N.R., Barakat R.R., Dickler M.N., Carter J. (2017). Sexual Health Needs and Educational Intervention Preferences for Women with Cancer. Breast Cancer Res. Treat..

[56] Cook E., Sutherland E., Baum G., Schover L., Newman L. (2017). Missing documentation in breast cancer survivors: Genitourinary syndrome of menopause. Menopause.

[57] Chin S.N., Trinkaus M., Simmons C., Flynn C., Dranitsaris G., Bolivar R., Clemons M. (2009). Prevalence and severity of urogenital symptoms in postmenopausal women receiving endocrine therapy for breast cancer. Clin. Breast Cancer.

[58] Crandall C., Petersen L., Ganz P.A., Greendale G.A. (2004). Association of breast cancer and its therapy with menopause-related symptoms. Menopause.

[59] Biglia N., Cozzarella M., Cacciari F., Ponzone R., Roagna R., Maggiorotto F., Sismondi P. (2003). Menopause after breast cancer: A survey on breast cancer survivors. Maturitas.

[60] Baumgart J., Nilsson K., Stavreus-Evers A., Kask K., Villman K., Lindman H., Kallak T., Sundström-Poromaa I. (2011). Urogenital disorders in women with adjuvant endocrine therapy after early breast cancer. Am. J. Obstet. Gynecol..

[61] American College of Obstetricians and Gynecologists’ Committee on Clinical Consensus—Gynecology (2021). Treatment of Urogenital Symptoms in Individuals with a History of Estrogen-dependent Breast Cancer: Clinical Consensus. Obstet. Gynecol..

[62] Biglia N., Bounous V.E., D’Alonzo M., Ottino L., Tuninetti V., Robba E., Perrone T. (2017). Vaginal Atrophy in Breast Cancer Survivors: Attitude and Approaches Among Oncologists. Clin. Breast Cancer.

[63] Rabley A., O’Shea T., Terry R., Byun S., Louis Moy M. (2018). Laser Therapy for Genitourinary Syndrome of Menopause. Curr. Urol. Rep..

[64] Stefano S., Stavros A., Massimo C. (2015). The use of pulsed CO_2_ lasers for the treatment of vulvovaginal atrophy. Curr. Opin. Obstet. Gynecol..

[65] Franić D., Fistonić I. (2019). Laser Therapy in the Treatment of Female Urinary Incontinence and Genitourinary Syndrome of Menopause: An Update. BioMed Res. Int..

[66] Salvatore S., Leone Roberti Maggiore U., Athanasiou S., Origoni M., Candiani M., Calligaro A., Zerbinati N. (2015). Histological study on the effects of microablative fractional CO_2_ laser on atrophic vaginal tissue: An ex vivo study. Menopause.

[67] Zerbinati N., Serati M., Origoni M., Candiani M., Iannitti T., Salvatore S., Marotta F., Calligaro A. (2015). Microscopic and ultrastructural modifications of postmenopausal atrophic vaginal mucosa after fractional carbon dioxide laser treatment. Lasers Med. Sci..

[68] Lapii G.A., Yakovleva A.Y., Neimark A.I. (2017). Structural Reorganization of the Vaginal Mucosa in Stress Urinary Incontinence under Conditions of Er:YAG Laser Treatment. Bull. Exp. Biol. Med..

